# Susac Syndrome and Role of Therapeutic Plasma Exchange: A Case Report

**DOI:** 10.7759/cureus.48811

**Published:** 2023-11-14

**Authors:** Salem Vilayet, Anand Achanti, Munsef Barakat, Milos Budisavljevic, Tibor Fulop

**Affiliations:** 1 Department of Medicine, Division of Nephrology, Medical University of South Carolina, Charleston, USA

**Keywords:** anti-gad 65 encephalitis, central nervous system vasculitis, autoimmune encephalopathy, therapeutic plasma exchange (tpe), susac syndrome

## Abstract

Susac syndrome is a relatively uncommon autoimmune disease that predominantly affects young females, with the highest incidence between the third and fourth decade of life, presenting classically with encephalopathy, various CNS dysfunctions, visual impairment due to retinal artery occlusion, and hearing loss. Despite treatment options, such as glucocorticoid steroids, intravenous immunoglobulin, methotrexate, azathioprine, mycophenolate mofetil, or rituximab, some patients with Susac syndrome remain refractory to therapy. We present a case report of a 38-year-old female with refractory Susac syndrome who was treated successfully with plasmapheresis.

## Introduction

Susac syndrome is a rare autoimmune endotheliopathy that predominantly affects young females with female:male ratio 3:1, peak incidence between 20 and 40 years, clinical presentation is variable and a classical triad of encephalopathy or focal CNS dysfunction, headache, visual dysfunction from retinal artery occlusion, and sensorineural hearing loss presents in <15% of cases [1-3].

Other symptoms could include psychiatric symptoms, personality changes, and even seizures. Diagnosis can be challenging, especially in the absence of a classic triad, and the main differential diagnosis is stroke and other intracranial pathology. Neuroimaging, especially MRI of the brain, can be suggestive. Treatment of Susac syndrome largely depends on the extent and severity of the presentation. Treatment options include steroids, intravenous immunoglobulins (IVIGs), immunosuppressant agents, and therapeutic plasma exchange (TPE). We present a case of Susac syndrome that was refractory to immunosuppression and treated successfully with plasmapheresis. Part of this article and the case presentation were previously presented at the American Society for Apheresis 44th Annual Meeting, April 26-28, 2023 [3].

## Case presentation

**Figure 1:**
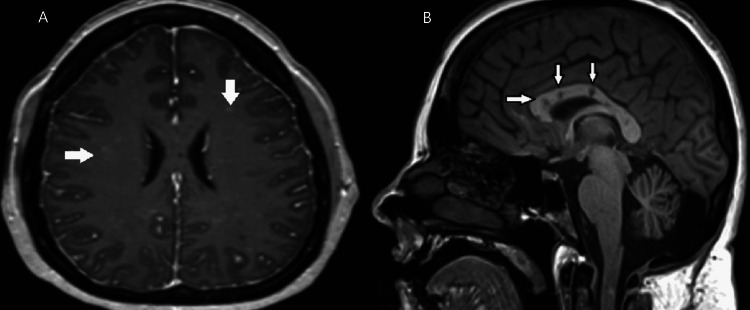
Axial (A) and sagittal (B) T1-weighted magnetic resonance image demonstrating central callosal punched-out lesions (white arrows)

A 38-year-old Caucasian female with a history of Susac’s syndrome, anxiety, and depression presented to the emergency provider with confusion, aphasia, and headache. The patient was diagnosed with Susac’s syndrome about four months prior to this visit. She had initially presented with persistent vomiting and headaches subsequently MRI of the brain showed multifocal T1 hypointensities along corpus callosum lesions concerning vasculitis (Figures 1A, 1B). Her brainstem auditory evoked response showed dysfunction of the distal auditory nerve on the right. Subsequently underwent lumbar puncture which was unremarkable except for lymphocytic pleocytosis with positive serum GAD65. Funduscopic examination is notable for a cotton wool spot on the left eye.

She started on high-dose methylprednisolone (IV of 1 g daily) and transitioned to oral prednisone with an outpatient taper regimen. Due to a subsequent deterioration, the IV methylprednisolone course was repeated and started on mycophenolate mofetil, along with IVIG q2 weeks. The patient continued to have ongoing headaches along with periods of confusion and was subsequently brought back to the hospital. She was noted to have new expressive aphasia which did not improve despite being started on IV pulse dose steroids x3 days; repeat MRI of the brain was remarkable for multiple T2/Flare hyperintense foci throughout the supratentorial cerebral hemisphere, with interval development of diffuse multifocal parenchymal and leptomeningeal enhancement concerning for disease progression. A decision was made to initiate TPE using Spectra Optia continuous-flow centrifugal apheresis system using 5% albumin as replacement every other day for five sessions with a centrifugal device, achieving resolution of aphasia and headache, enabling discharge home with an outpatient regimen of prednisone and MMF.

## Discussion

Susac syndrome is thought to be caused by autoreactive cytotoxic CD8+ T cells causing inflammation-mediated injury to the microvascular endothelium, causing micro-ischemic damage. The disease course is often self-limited with a monocyclic (self-limited disease after a maximum period of two years), polycyclic (fluctuating disease self-limited to a maximum of two years), or chronic-continuous course (lasting more than two years) [1]. The patients are generally treated with glucocorticoid steroids during the acute phase and, in severe cases, immunotherapy with IVIG, methotrexate, azathioprine, mycophenolate mofetil, or rituximab [1].

The rate-limiting enzyme involved in converting glutamate to GABA, the primary inhibitory neurotransmitter in the central nervous system, is known as glutamic acid decarboxylase (GAD). In various neurological disorders such as seizures, stiff-man syndrome, cerebellar ataxia, epilepsy, and encephalitis, antibodies targeting GAD have been identified as a potential factor. Anti-GAD antibodies have two major isoforms, GAD65 and GAD67. The presence of these antibodies may lead to treatment resistance to standard medical therapy, like in our patient [4,5].

TPE is an extracorporeal treatment that non-selectively removes all plasma proteins and replaces them with albumin. It is an emerging modality in the treatment of patients with various neurologic and non-neurologic diseases [6]. Although the use of TPE has been reported in Susac syndrome, its role and the timing of its use are not well established. In our case, the decision to start TPE was based on a lack of response to initial treatment with steroids and immunosuppressants, along with clinical and radiological evidence of disease progression.

## Conclusions

Susac syndrome is a rare disorder, and a high degree of clinical suspicion is needed for an accurate diagnosis. It is characterized by relapsing and remitting active inflammatory episodes, and although complete remission has been reported, most patients continue to have chronic inflammation that can have serious neurological sequelae. Treatment of active relapse is essential to control symptoms and reverse ongoing damage. TPE should be considered in cases of severe and refractory Susac patients and those with positive GAD 65 autoantibodies.
